# Verifiability of diagnostic categories and work ability in the context of disability pension award: A survey on "gatekeeping" among general practitioners in Norway

**DOI:** 10.1186/1471-2458-8-137

**Published:** 2008-04-25

**Authors:** Rein Overland, Simon Overland, Kristian Nyborg Johansen, Arnstein Mykletun

**Affiliations:** 1 Research centre for Health Promotion, University of Bergen, Norway; 2 Norwegian Institute of Public Health, Division of Mental Health, Oslo, Norway

## Abstract

**Background:**

Disability benefits exist to redeem social and financial consequences of reduced work ability from medical conditions. Physicians are responsible for identifying the medical grounds for benefit claims. The aim of this study was to explore physicians' views on verifiability of medical conditions and related work ability in this context.

**Methods:**

Information on verifiability of diagnostic categories and work ability was obtained from a survey among a representative sample of general practitioners (GPs) in Norway (n = 500, 25.2% response rate). Verifiability was defined as to what extent the assessment is based on objective criteria versus on information from the patient. We enquired about the diagnostic categories used in official statistics on main disability benefit causes in Norway and elsewhere.

**Results:**

On a scale from 0 (low verifiability) to 5 (high verifiability), the mean level of verifiability across all diagnostic categories was 3.7 (SD = 0.42). Degree of verifiability varied much between diagnostic categories, and was low in e.g. unspecified rheumatism/myalgia and dorsopathies, and high in neoplasms and congenital malformations, deformation and chromosomal abnormalities. Verifiability of work ability was reported to be more problematic than that of diagnostic categories. The diagnostic categories rated as the least verifiable, are also the most common in disability pension awards.

**Conclusion:**

Verifiability of both diagnostic categories and work ability in disability assessments are reported to be moderate by GPs. We suggest that the low verifiability of diagnostic categories and related work ability assessments in the majority of disability pension awards is important in explaining why GPs find the gatekeeping-function problematic.

## Background

The disability pension scheme is developed to help those who cannot generate a livelihood through paid work due to medical reasons. The medical certification and assessment of work ability from the present medical conditions is usually handled by physicians, most often by family doctors or general practitioners (GPs). In this context, the physician must determine the correct diagnosis (one or two) causing the reduced work ability, and also whether work ability is reduced to an extent that a disability pension is warranted. This function implies finding a balance between advocating the patients' opinions and needs, and acting as a gatekeeper on behalf of society by restricting access to public benefit-schemes [1].

According to formalities, the national insurance administration has the final say in applications for disability pension. However, the physicians' medical opinions are likely to be highly weighted and may often be the in-effect gatekeeping function [2]. The execution of this function is difficult when objective criteria of a disease are scarce.

In most of the Organization for Economic Co-Operation and Development (OECD) countries, there has been a steady increase in the rate of the working-age population who becomes recipients of disability pensions prior to age retirement [3]. There is a growing concern that the rise in disability expenditure is a grave fiscal threat for existing welfare systems [4].

Norway is among the countries with the highest inflow to disability benefits schemes (comprising disability pension, sick-leave, rehabilitation and vocational training) among OECD members [3]. By the end of 2006, 11% of the working age population in Norway were recipients of disability pension [5]. In official statistics, mental and behavioural disorders and disorders of the musculoskeletal system and connective tissue are the most frequently used diagnostic categories warranting disability pension. These two categories account for nearly 2/3 of disability pensions, as of 2004. Beyond these, other frequent categories are diseases of the nervous system (6.7%) and diseases of the circulatory system (10.8%) [6].

Despite the important role of physicians in the context of disability assessment and certification, only a few studies have focussed on work ability assessments [1]. Physicians find such assessments difficult and think there is a lack of training in this task [7]. Their assessments varies to a large extent [8], an observation that is supported from case-vignette studies showing discrepancies in GPs' assessed need for disability benefit [9]. Part of being involved in certification, is being a gatekeeper. This role was in a study among UK GPs perceived as in conflict with maintaining an adequate doctor-patient relationship. Most participants emphasized responsibility to their patient as their main focus, thereby outweighing the gatekeeping-role assigned by the authorities [10].

In Norway, a recent reform (2001) entitled all citizens to have a designated GP. These GPs are obliged to prioritise patients on his or her list, and receive a fixed compensation per listed patient. Patients are allowed to switch GP twice a year [11]. Recent qualitative studies suggest that this reform has made physicians less engaged in gatekeeping practices, partly due to economic incentives to keep patients on their lists [12].

According to a recent review, physicians report problems with sick-listing and with the issuance of medical certificates for disability pensions [1]. In particular, difficulties in determining duration of sick-leave, establishing the correct diagnosis, determining the validity of the patients' presented case, and having patients follow the physicians' advice was underlined [13]. Physicians report finding it difficult to assess work ability [7,13], and they differ in their judgements regarding intervention and rehabilitation [14].

The current benefit systems are conditional upon GP's ability to make the correct diagnosis and associated work ability. Studies mentioned above suggest that physicians find this task challenging. We wanted to explore physicians' opinions on how verifiable medical conditions and related work ability are in the context of disability pension award.

## Methods

We distributed a questionnaire to a random five hundred GPs registered in the Norwegian Labour and Welfare Organisation (Additional files 1 &2). The survey was distributed evenly to GPs across Norwegian counties. No demographic information on the GPs was obtained to ensure anonymous responses.

To collate feasible and relevant diagnostic categories, the questionnaire contained a list of the seventeen most frequent diagnostic categories in disability pension award in 2004, following the categories employed by the National Insurance Administration [6]. Main diagnostic categories (e.g. injury, poisoning and certain other consequences of external causes) were preferred when subcategories equalled less than 1.6%. Consequently, we included some minor diagnostic categories (e.g. diseases of the skin and subcutaneous tissue) on the expense of others (e.g. endocrine, nutritional and metabolic diseases).

We asked the GP's to give their opinion on verifiability of these diagnostic categories, and also verifiability of reduced work ability caused by these. Verifiability was thus defined in context of the GP as the gatekeeper, responsible for identifying the medical grounds for benefit claims. Verifiability was also used as a term in our scale: Responses were collected applying a six-point Likert scale with scale extremes labelled "The diagnosis is set based on biomarkers or other objective criteria" (high verifiability) versus "The diagnosis is set based on information from the patient" (low verifiability). Correspondingly, degree of verifiability of work ability (as resulting from the diagnosis) was measured with the scale extremes "Degree of work ability is decided based on biomarkers or other objective criteria" (high verifiability) versus "Degree of work ability is decided based on information from the patient" (low verifiability). The questionnaire is available as Appendix 1.

To avoid demand characteristics, the presented aim of the study was limited to "exploring health insurance issues". A subsection of the law on disability pension access was quoted as a reminder of the medical professionals' mandate in this regard. Participants were informed that international studies suggest that physicians in some cases find assessment of diagnosis and work ability challenging in relation to disability benefit (Appendix 1 for details).

No reminders were sent out, and no incentive beyond receiving the present paper was offered to the participants. Of 126 valid responders, 109 were fully complete, nine had missing data for one item only, and the remaining eight had missing data on four or fewer items (of 34 items, 17 for diagnostic categories, and 17 for related work ability). No diagnostic categories were in particular subject to missing responses. All 126 survey responders were included in the analyses, and no attempt to replace missing data was done.

The results were analyzed using Statistical Product and Service Solutions 14.0.1 (SPSS). Means were calculated with a 95% confidence interval, and a paired sample t-test was applied to test of statistical significance.

## Results

On average, a moderate level of verifiability of both diagnostic categories and work ability was found. On the six-point scale from 0 (low verifiability) to 5 (high verifiability), the overall mean was 3.7 (SD = 0.42) for diagnostic categories and 3.0 (SD = 0.52) for work ability. Diagnostic categories was in general reported to be easier to verify than work ability (p < .01).

Although there are large variations in mean scores on the variables, few respondents have used the extreme values on the scale. On the higher end of the scale, only 3% of GPs had an individual mean score greater than 4.5 across the seventeen diagnostic categories regarding verifiability. On the low end, only 1% had an individual mean score less than 2.5. For work ability, no GP had an individual mean score greater than 4.5, and 16% of the respondents had an individual mean score lower than 2.5.

**Figure 1:**
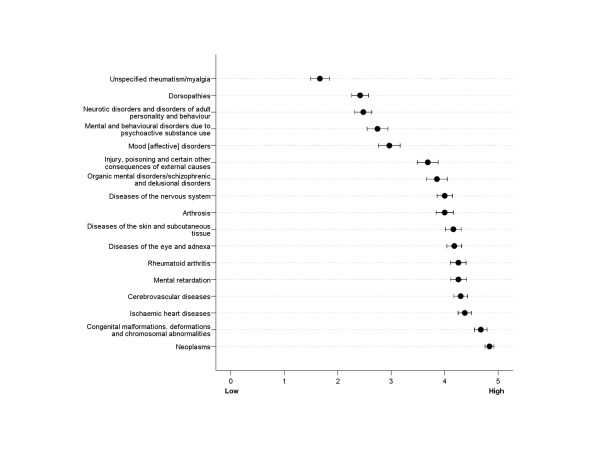
Verifiability of diagnostic categories, 95% confidence intervals.

**Figure 2:**
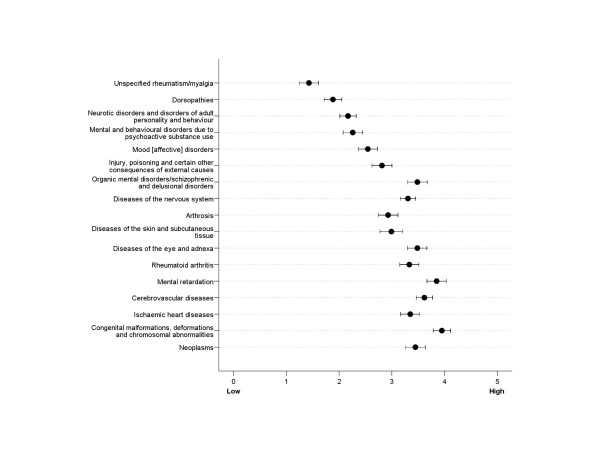
Verifiability of work ability, 95% confidence intervals.

Verifiability varied much across diagnostic categories (Figure 1). Across the GPs, neoplasms (mean = 4.8) were reported to be the most verifiable, followed by congenital malformations, deformations and chromosomal abnormalities (mean = 4.7) and ischaemic heart diseases (mean = 4.4). The least verifiable diagnostic categories were unspecified rheumatism/myalgia (mean = 1.7), dorsopathies (mean = 2.4), and three categories in the mental illnesses spectrum (means within range 2.5 to 2.9). A similar pattern was found for work ability resulting from these categories (Figure 2), but verifiability of work ability was generally regarded more difficult than diagnostic categories (all p < .001).

**Figure 3:**
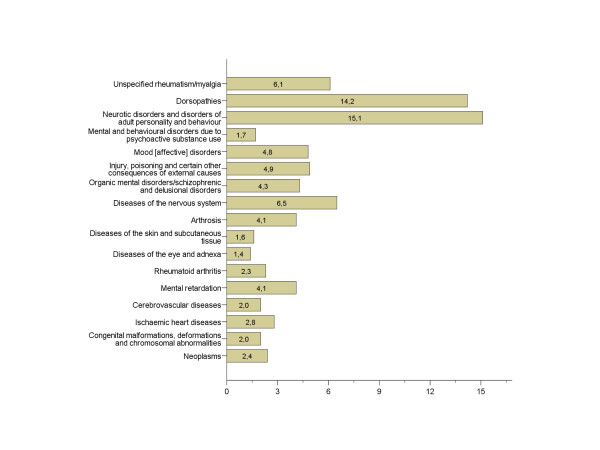
Percentage of diagnostic categories warranting disability pension awards in 2004 (regardless of year of award).

The three diagnostic categories, which are reported to be the least verifiable, are all among the top five most frequent categories warranting disability pension award in Norway. Altogether they comprise 35.4% of awarded disability pensions (Figure 3). The three most verifiable diagnostic categories comprise 7.2% of awarded disability pensions. The same pattern was found regarding verifiability of work ability resulting from these conditions, although the most verifiable conditions differed to some extent.

## Discussion

### Strenghts and limitations

In all essence, general medical care is in Norway provided by General Practitioners (GPs) who receive public funding. Delivery of general medical care in private practices outside this arrangement is marginal (probably delivering less than 1% of services). The participants in this study were drawn from GPs receiving public funding, ensuring a highly representative sample for Norwegian GPs. However, faced with a low response rate, systematic participation bias is a concern. In this form of bias, the biased findings would necessarily be avoided if everyone chose to participate. In the present study, GPs reported that some diagnostic categories (e.g. neoplasms) are more "objectively" defined than others (musculoskeletal disorders) in the context of disability pension award. For this result to be a product of response bias there would need to be large, systematic differences of opinion between respondents and non-respondents: Non-responders would to a much larger extent need to hold the opposite or neutral opinion (e.g. that cancer is less or just as objectively verifiable as musculoskeletal disorders), which we find unlikely to be the case.

Our other main finding is that diagnoses are more verifiable than the related work ability (also in the context of assessment for disability pension award). Using the same line of argument as above, we think it is unlikely that the non-participants would have so different opinions that their non-participation was the sole reason for our findings.

Based on this, we do acknowledge that the low response rate is a major concern, but we do not believe that our two main findings merely to be a product of bias from selective participation.

We used diagnostic categories according to those used in official statistics. The diagnostic categories are quite broad, and as a result comprise diagnoses where verifiability varies within. This limitation will most likely reduce variance between the diagnostic categories.

**Figure 4:**
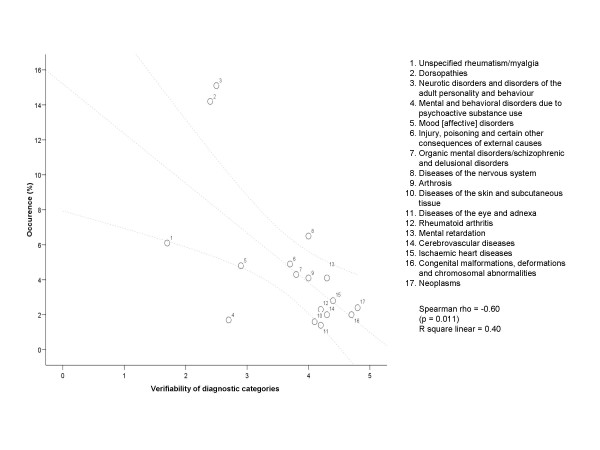
Diagnostic categories with low verifiability often occur as official diagnoses for disability pension award.

The inclusion of both main diagnostic categories and subcategories has implications for our results, as the occurrence will be somewhat misleading. This limitation is especially relevant for Figure 4, as we illustrate a negative association between verifiability and occurrence. In e.g. diseases of the circulatory system as a main category includes both ischaemic and cerebrovascular diseases and has an overall occurrence of 7%. Any categorising of diagnoses has its limitations, as does also the official categorization by the National Insurance Administration we have employed [6].

A core assumption in the present study is that GPs opinions of verifiability are relevant for their actual practice and behaviour in cases of disability assessment. This cannot be fully answered from the present study. However, we cannot see how gatekeeping practices can be any more reliable than reflected in the GPs opinions on verifiability of various conditions.

Our scales (and the entire study) rest on two presumptions. First; health problems and related work ability do vary in terms of how verifiable they are. Second; information obtained in dialogue with the patient is less verifiable than information based on biomarkers. Our findings are no more valid than these presumptions. As such, a major limitation of our study is that the value and importance of clinical judgement is not incorporated.

### Interpretation

The three diagnostic categories, which are reported to be the least verifiable, are all among the top five most frequent categories warranting disability pension award in Norway (Table 1). This underlines the challenges physicians face regarding issuance of disability pension certificates and role as gatekeepers [7-10]. The same pattern was found for verifiability of work ability in these conditions. Approximately 2/3 of disability pensions are awarded for a diagnosis largely based on information from the patient. The lack of biomarkers/objective criteria in the most common diagnostic categories for which disability pensions are awarded, might contribute to the conflict between the GPs' patient advocacy and gatekeeping roles.

**Table 1:** Verifiability of diagnostic categories and work ability, and occurrence of diagnostic categories warranting disability pension awards

Diagnostic categories descending by verifiability of diagnostic categories	Diagnostic categories † mean (SD)	Work ability ‡ mean (SD)	Occurrence *
Unspecified rheumatism/myalgia	1.7 (0.95)	1.4 (0.98)	6.1
Dorsopathies	2.4 (0.84)	1.9 (0.87)	14.2
Neurotic disorders and disorders of adult personality and behaviour	2.5 (0.84)	2.2 (0.81)	15.1
Mental and behavioural disorders due to psychoactive substance use	2.7 (1.05)	2.3 (0.97)	1.7
Mood [affective] disorders	2.9 (1.07)	2.5 (0.96)	4.8
Injury, poisoning and certain other consequences of external causes	3.7 (1.06)	2.8 (1.04)	4.9
Organic mental disorders/schizophrenic and delusional disorders	3.8 (1.03)	3.5 (0.99)	4.3
Diseases of the nervous system	4.0 (0.76)	3.3 (0.77)	6.5
Arthrosis	4.0 (0.87)	2.9 (0.98)	4.1
Diseases of the skin and subcutaneous tissue	4.1 (0.82)	2.9 (1.13)	1.6
Diseases of the eye and adnexa	4.2 (0.74)	3.5 (0.97)	1.4
Rheumatoid arthritis	4.2 (0.77)	3.3 (0.93)	2.3
Mental retardation	4.3 (0.79)	3.9 (0.82)	4.1
Cerebrovascular diseases	4.3 (0.68)	3.6 (0.97)	2.0
Ischaemic heart diseases	4.4 (0.68)	3.4 (0.94)	2.8
Congenital malformations, deformations and chromosomal abnormalities	4.7 (0.62)	4.0 (0.87)	2.0
Neoplasms	4.8 (0.45)	3.4 (0.98)	2.4

† Verifiability of diagnostic categories as reported in survey. Range 0 (low) to 5 (high)

‡ Verifiability of work ability as reported in survey. Range 0 (low) to 5 (high)

* Percentage of diagnostic categories warranting disability awards in 2004 according to official statistics (regardless of year of award)

According to Norwegian law regulating disability pensions, the following criteria must be met for eligibility; " [...] the person concerned has a lasting disease, injury or defect [...] A conception of disease which is scientifically based and commonly approved in medical practice shall form the basis when the occurrence of a disease is being considered [...] Social or economic problems do not entitle benefits according to this chapter [...] The medical condition must cause a lasting reduction of work ability to such a degree that it is the main reason to reduced earning potential/work ability" [15]. Similar criteria for access to disability pension are emphasized in other countries [1]. The key information requested has always been related to the patients' diagnosis and the loss of work ability resulting from this diagnosis. When biomarkers or objective criteria are scarce, it is possibly harder to know whether the conclusions fully conform to the abovementioned directives.

Our results suggest that the directive concerning work ability is the most challenging part of the GPs disability assessment. As reduced work ability is the key factor for issuing benefits, this result further underlines the difficulties inherent in disability assessments. These findings might serve to explain previous findings where work ability amongst disability benefit recipients, only partially could be attributed to symptoms and diagnoses [16].

Physicians' perceptions of patients' medical needs are reported to be stronger predictors of the physicians' behaviours in consultations than the patients own preferences measured prior to the consultation [17]. The authors stress the importance of eliciting the patient expectations to limit unnecessary use of resources and iatrogenesis. Based on such findings, we speculate that when verifiability of diagnostic categories and work ability are low for common diagnoses in disability pension award, examinations and decision-making must be highly dependant of the GPs perception and interpretation of symptoms and work ability as reported by the patient. Case-vignette studies have shown that the assessment of, and the perceived need for disability benefits, varies among physicians [8,9]. Such individual differences have implications for the concept of fair and legitimate resource allocation.

According to a recent report by OECD [3], the disability program in Norway has several challenges as the disability pensioning increase without a corresponding deterioration of general health in the population [18-20]. In a similar fashion, there is little evidence to suggest an increase in the true prevalence of mental illnesses [21] and musculoskeletal [22,23] disorders that compose the diagnostic bulk of benefits. These findings call for explanations beyond health to explain the increase of disability pension recipients in Norway.

The conundrum of the increase in disability pension recipients despite improvement in key health indicators of the western working-age population has inspired rational choice models, often labelled as "pull models". These models explain the transition from work to disability pension award as a result of rational considerations of pros and cons in the two alternatives, taking both economy and leisure time into account [24]. In accordance with this model, people are rational actors seeking to maximize utility for their own good. Utility is defined as a function of economy and leisure time. According to this model, the GP's power in the gatekeeping role is marginal, and the model does seem to presume that the patient will access disability benefits if motivated to apply. There is some empirical support for this model; In Canada and Quebec (Quebec has its own pension plan), the effect of an increase in disability benefit salary on the composition of the medical conditions on the disability roll was studied. An increased payment in disability schemes (about $150 a month) in Canada resulted in an increase of incidence of hard-to-diagnose conditions (musculoskeletal disorders). In Quebec, where no such increase was implemented, the incidence of hard-to-diagnose conditions was fairly stable in the same period [25]. Our study does not provide evidence neither for or against pull model predictions, but the common presence of disability pensioning with diagnoses with low verifiability may partly be a result of patient strategies as postulated by the rational choice model [24].

In contrast to the pull-model, the push-model focuses on involuntary factors beyond the individual, forcing the employee out of work and on to disability benefit arrangements. Such factors can be work-place characteristics and/or economic structures in the society [26]. Profit needs, effectiveness criteria, and the pace of work place changes might exclude certain individuals from the labour marked. There is empirical support for the hypothesis that disability benefit is used for early retirement in such cases [27]. Krokstad & Westin conclude that medical determinants alone cannot explain the increased incidence rates of disability pension, and underline the importance of social, non-medical, and contextual determinants' in disability [28].

The process of moving people on to disability benefit as a consequence of structural problem of the labour market and society is commonly known as a process of medicalization. In this perspective, the individual is forced to obtain a medical diagnosis and demonstrate impaired work ability to be granted a disability benefit. The high frequency of diagnostic categories with low verifiability might thus be a result of push-factors and involuntary processes rather than pull factors and un-intended financially motivated responses to the welfare system.

Finally, the increase in mental and musculoskeletal diagnoses warranting disability benefits can be caused by changes in work demands, so that people with such health problems have a harder time retaining a job than before.

## Conclusion

In the context of disability benefit assessments, GPs in Norway find verifiability of diagnostic categories and work ability to be moderate. The degree of verifiability varies between categories. Work ability is found to be significantly harder to verify than diagnostic categories. This difference accounts for all categories included in the survey. The least verifiable diagnostic categories are the most frequent categories warranting disability pension awards.

Despite public health improvements on several parameters over recent decades, the amount of disability-benefit recipients is augmenting. As this cannot be explained by an increase of medical conditions, other explanations have been postulated. Pull-factors may attract individuals as a consequence of generous benefit arrangements. Push-factors in the work place and society may force individuals out of labour on to disability benefit schemes. Regardless of whether push- or pull-factors are in effect, part of the large proportion of diagnostic categories with low verifiability could be caused by people who cannot work, but do not conform fully with the medical emphasis in the disability pension scheme.

## Competing interests

The authors declare that they have no competing interests.

## Authors' contributions

RO participated in design of the study, data collection, drafted and continuously revised the manuscript according to comments of the co-authors, and carried out the data analysis. SO participated in design of the study, interpretation of results and revised the manuscript for important content. KNJ contributed to study design, data collection and data analysis. AM supervised during planning of the study, data collection and contributed in revising the manuscripts. All authors read and approved the final manuscript.

## Pre-publication history

The pre-publication history for this paper can be accessed here:



## Supplementary Material

Additional file 1 Survey of verifiability of diagnostic categories in relation to disability pension. The questionnaire in which the GPs gave their responses. Click here for file

Additional file 2 Survey among general practitioners regarding verifiability of diagnostic categories in questions of disability pension. Information-letter to the GPs describing the main aspect of the survey. Click here for file
