# Supplementary material for: Verifiability of diagnostic categories and work ability in the context of disability pension award: A survey on "gatekeeping" among general practitioners in Norway
Source: BMC Public Health. 2008 Apr 25;8:137. doi: 10.1186/1471-2458-8-137 (PMC2387147; doi:10.1186/1471-2458-8-137)
Supplement: Additional file 1 — Survey of verifiability of diagnostic categories in relation to disability pension. The questionnaire in which the GPs gave their responses. [file 1471-2458-8-137-S1.doc]

**Survey of verifiability of diagnostic categories in relation to disability pension**

Verifiability of diagnostic categories:

High: The diagnosis is made based on biomarkers or other objective criteria.

Low: The diagnosis is made based on information from the patient.

Verifiability of work ability:

High: Degree of impairment is decided based on biomarkers or other objective criteria.

Low: Degree of impairment is decided based on information from the patient.

|  |  | **Verifiability of**  **diagnosis** | |  | **Verifiability of work ability from the diagnosis** | |
| --- | --- | --- | --- | --- | --- | --- |
|  |  | **Low**  Based on information from the patient | **High**  Based on objective findings |  | **Low**  Based on information from the patient | **High**  Based on objective findings |
|  |  |  | |  |  | |
| Neoplasms |  | 0 1 2 3 4 5 | |  | 0 1 2 3 4 5 | |
| Organic mental disorders/schizophrenic and delusional disorders |  | 0 1 2 3 4 5 | |  | 0 1 2 3 4 5 | |
| Mood [affective] disorders |  | 0 1 2 3 4 5 | |  | 0 1 2 3 4 5 | |
| Neurotic disorders and disorders of adult personality and behaviour |  | 0 1 2 3 4 5 | |  | 0 1 2 3 4 5 | |
| Mental and behavioural disorders due to psychoactive substance use |  | 0 1 2 3 4 5 | |  | 0 1 2 3 4 5 | |
| Mental retardation |  | 0 1 2 3 4 5 | |  | 0 1 2 3 4 5 | |
| Diseases of the nervous system |  | 0 1 2 3 4 5 | |  | 0 1 2 3 4 5 | |
| Diseases of the eye and adnexa |  | 0 1 2 3 4 5 | |  | 0 1 2 3 4 5 | |
| Ischaemic heart diseases |  | 0 1 2 3 4 5 | |  | 0 1 2 3 4 5 | |
| Cerebrovascular diseases |  | 0 1 2 3 4 5 | |  | 0 1 2 3 4 5 | |
| Diseases of the skin and subcutaneous tissue |  | 0 1 2 3 4 5 | |  | 0 1 2 3 4 5 | |
| Rheumatoid arthritis |  | 0 1 2 3 4 5 | |  | 0 1 2 3 4 5 | |
| Arthrosis |  | 0 1 2 3 4 5 | |  | 0 1 2 3 4 5 | |
| Dorsopathies |  | 0 1 2 3 4 5 | |  | 0 1 2 3 4 5 | |
| Unspecified rheumatism/myalgia |  | 0 1 2 3 4 5 | |  | 0 1 2 3 4 5 | |
| Congenital malformations, deformations and chromosomal abnormalities |  | 0 1 2 3 4 5 | |  | 0 1 2 3 4 5 | |
| Injury, poisoning and certain other consequences of external causes |  | 0 1 2 3 4 5 | |  | 0 1 2 3 4 5 | |
