# Supplementary material for: Verifiability of diagnostic categories and work ability in the context of disability pension award: A survey on "gatekeeping" among general practitioners in Norway
Source: BMC Public Health. 2008 Apr 25;8:137. doi: 10.1186/1471-2458-8-137 (PMC2387147; doi:10.1186/1471-2458-8-137)
Supplement: Additional file 2 — Survey among general practitioners regarding verifiability of diagnostic categories in questions of disability pension. Information-letter to the GPs describing the main aspect of the survey. [file 1471-2458-8-137-S2.doc]

Dear [sirname]

Bergen 17.01.07

**Survey among general practitioners regarding verifiability of diagnostic categories in questions of disability pension**

We address you in relation to a survey regarding clinical assessments in questions of disability pension. The survey makes part of a broader scientific project exploring health insurance issues. Your contact-details were gathered through the National Insurance Agency’s lists displaying GPs. The survey is sent to 500 GPs, distributed in all of the 19 counties.

The access to disability pension is regulated through the law of Social Services, § 12-6:

*“§ 12-6. Disease, injury or defect – need for causality*

*To be eligible for benefits according to this chapter, it is required that the person concerned has a lasting disease, injury or defect.*

*A conception of disease, which is scientifically based and commonly approved in medical practice, shall form the basis when the occurrence of a disease is being considered. Social or economic problems do not entitle benefits according to this chapter.*

*The medical condition must cause an enduring impairment of work ability to such a degree that it is the main reason to reduced earning potential/work ability.”*

Some diagnoses (*disease, injury or defect*) can be set due to objective findings. However, other diagnoses require more clinical judgements in the presence of subjective symptoms.

The GP are also to assess if the diagnosis is leading to an enduring impairment. International studies suggest that GPs, in some cases, find these assessments challenging.

We are grateful if you spare some time answering our survey and return it before 30.01.07. The information will be treated confidentially. If you e-mail us on the address [trygdeforskning@uib.no](mailto:trygdeforskning@uib.no), we will send you the publication.

Best regards

Arnstein Mykletun, Phd
